# Disciplinary issues in special education: Teachers’ experiences and attribution

**DOI:** 10.4102/ajod.v14i0.1655

**Published:** 2025-09-19

**Authors:** Mmanako F. Mokano, Motsekiso C. Letuma

**Affiliations:** 1 Department of Education Management, Policy and Comparative Education, Faculty of Education, University of Free State, Qwaqwa, South Africa; 2 Department of Education Management, Policy and Comparative Education, Faculty of Education, University of Free State, Bloemfontein, South Africa

**Keywords:** classroom management, disability, indiscipline, special education, secondary schools

## Abstract

**Background:**

Special education facilities are designed to support learners with special needs. These efforts are vital for ensuring inclusive, equitable education and promoting lifelong learning, which aligns with the Sustainable Development Goals. However, a positive learning environment, including addressing disciplinary issues, is necessary for learning.

**Objectives:**

This study explored the disciplinary issues that teachers experience in special schools and the contributing factors.

**Method:**

The study adopted an interpretive paradigm and employed a qualitative methodology using a phenomenological design. It was grounded on Bronfenbrenner’s Ecological Systems Theory. The data were collected through semi-structured interviews with six special school teachers from two purposely selected secondary special schools and the data were analysed using inductive content analysis.

**Results:**

Teachers in special schools attributed learner indiscipline to disengagement, absenteeism, and bullying. These behaviours were linked to a variety of factors such as curriculum pressure, teacher leniency, family dynamics, communication barriers, social grant support, racial influences, peer influence and disability-related behaviour.

**Conclusion:**

A holistic approach that includes enhancing teacher training to equip educators with effective classroom management strategies and strengthening home-school partnerships through regular communication and parenting workshops to support family dynamics and address behavioural concerns is recommended. Moreover, training for adapting the curriculum to be more inclusive and diverse learning, as well as establishing peer mentoring programmes to encourage positive behaviour and reduce bullying through social support, are recommended.

**Contribution:**

The study contributes to the knowledge surrounding indiscipline in special schools and opens further options for contribution to measures that could be implemented.

## Introduction

Children are not born with the same abilities or aptitudes. While every child requires some support, the level of assistance needed can vary significantly because of each child’s unique traits. Those who require more support than their peers are often referred to as children with special needs (Department of Basic Education [DBE] 2014). According to Krämer, Möller and Zimmermann (2021), these children require individualised, practical support to foster academic and social development. Thus, special education facilities are designed to provide this support (Phala & Hugo 2022). Such efforts are crucial in ensuring inclusive and equitable quality education and promoting lifelong learning opportunities, key components of the Sustainable Development Goals. However, a conducive learning environment must be established for learning to be effective. This means special schools should address indiscipline to create a safe and supportive atmosphere for all learners.

While scholars have not explicitly defined the term indiscipline, they have conceptualised it implicitly as the opposite of discipline, a concept widely examined within the school context. Drawing from scholars such as Potokri and Lumadi (2025), Ama, Moorad and Mukhopadyay (2020), Wolhuter and Van Der Walt (2020) and Segalo and Rambuda (2018), discipline is presented as a fundamental principle in schools that underpins the teaching and learning process. The literature highlights discipline as essential for fostering order, fairness, learner protection, regulation of behaviour among both learners and educators, and the promotion of social cohesion and safety. Weak discipline is often linked to the absence of shared ethical values and a lack of caring concern, empathy, and moral awareness (Ama et al. 2020). Within public and private institutions, discipline remains a cornerstone for the effective functioning of classrooms (Segalo & Rambuda 2018). Consequently, indiscipline, although not always directly defined, is inferred through behaviours that contradict these principles. It is widely recognised as a serious concern in schools because of its disruption of effective teaching and learning (Letuma 2024).

Several studies in the South African context, including those by Sumbane et al. (2023), Mahlaule, McCrindle and Napoles (2024), and Chirowamhangu (2024), have explored the challenges that stakeholders face in special schools. However, none of these studies specifically explored the issue of indiscipline within these schools. Research has highlighted that the unique characteristics of learners in special schools can lead to behavioural challenges, which, in turn, may, directly and indirectly, disrupt the learning environment (Cheng & Toran 2022; Shoko 2024). This suggests that children with special needs often exhibit behavioural issues that affect teachers’ ability to manage the classroom and create a conducive teaching and learning atmosphere, frequently because of their condition. This background forms the basis upon which the study is grounded. In addition, the study was sparked by recognising a biased emphasis in the literature about indiscipline, predominantly centred on mainstream schools, which significantly constrains exploring this issue within special school contexts. The article aims to contribute by enhancing the understanding of indiscipline in special schools.

In South Africa, learners diagnosed with disabilities such as learning disorders, visual impairments or blindness, hearing impairments or deafness, attention deficit hyperactivity disorder (ADHD), autism spectrum disorder (ASD), and intellectual disabilities (ID) are typically referred to special schools. However, before such referrals are made, a systematic process is followed by the Screening, Identification, Assessment and Support (SIAS) Policy (DBE 2014). This policy offers a standardised framework for identifying, assessing, and implementing appropriate support interventions for learners requiring additional assistance to enhance their inclusion and participation in the school environment. It aims to improve access to quality education for vulnerable learners who face a range of barriers to learning, whether in mainstream or special school settings (DBE 2014). These barriers may include family disruptions, language challenges, the effects of poverty, and various cognitive or physical impairments.

The SIAS process is supported by formal documentation and is implemented by educators in collaboration with school-based support teams (SBSTs) and district-based support teams (DBSTs). Learners’ support needs are classified as low, moderate, or high, based on the intensity and frequency of intervention required. This classification informs decisions regarding where the learner will be supported and the responsible personnel. The policy outlines five critical areas of support: the involvement of specialist personnel; access to assistive devices, specialised equipment, and learning support materials; curriculum differentiation to meet individual learner needs; ongoing professional development, including training, orientation, mentoring, and guidance; and improvements in environmental access, which may be provided temporarily rather than continuously (DBE 2014). The nature and extent of the required support to overcome learning barriers are determined by evaluating the current resources available to the learner and the school, the additional support still required, and the resources accessible at the provincial or district level.

Placement at a special school is only contemplated after all other assistance options at the local school have been thoroughly examined (DBE 2014). This indicates that, depending on the procedures followed, the ultimate choice to enrol learners in special schools in South Africa demonstrates that such a child genuinely merits placement in that institution.

Studies have shown that behavioural difficulties seen in educational settings, particularly in special education, are frequently associated with the intrinsic conditions of the learners (Cheng & Toran 2022). This suggests that occurrences of indiscipline in special schools may mainly arise from disability-related behaviours, which are closely and directly linked to the learners’ identified problems (Grigorenko et al. 2020). A continual concern relates to educators’ lived experiences and their interpretations or attributions of such behaviours.

## Literature review

### Bullying

Bullying remains a widespread form of indiscipline among children and adolescents and is widely recognised as both a public health concern and a significant risk factor for mental health issues (Abregú-Crespo et al. 2024). Using data from over 90 000 US public schools, Gage et al. (2021) found that bullying was reported across all establishments, particularly targeting learners based on disability, race, and gender. The study further found that Hispanic learners, black learners, and learners with disabilities faced higher rates of victimisation and disciplinary actions than their peers without disabilities. Similarly, Robinson et al. (2023) reported that 21.6% of learners with disabilities experienced significant bullying, compared to 14.5% of learners without disability, often resulting in psychological distress and academic struggles. Mareš (2018), through a meta-analysis of 121 studies from Euro-American contexts, observed that learners with disabilities are frequently ridiculed and bullied in primary and secondary schools. Ochi et al. (2020) found that bullying was strongly linked to early school refusal among children with ASD, underscoring the need for early identification and intervention. Mokoena (2024), through a qualitative study in Free State, South Africa, found that while bullying is acknowledged in schools, it is often concealed by victims, complicating intervention by school management teams. Collectively, these studies, spanning mainstream and special schools across global contexts, highlight bullying as a persistent and global challenge in education.

### Absenteeism

Absenteeism, broadly defined as a student’s failure to attend school for any reason, remains a pressing educational concern that disrupts the smooth running of school activities, particularly among learners with disabilities (Kearney 2016, as cited in Sasso & Sansour 2024). Simerly (2021) highlights that although educational authorities have implemented various strategies to curb absenteeism, learners with disabilities consistently face greater challenges in maintaining regular attendance. Through a comprehensive analysis in England, Lereya et al. (2023) applied multilevel regression models to examine the interplay between absenteeism, special educational needs (SEN), and student characteristics. Their findings indicated that learners with disabilities, especially those with emotional, behavioural, or social challenges, were disproportionately affected by high absence rates. Similarly, the United States Department of Education (2016, cited in Anderson 2021) reported that learners with disabilities are 1.4 to 1.5 times more likely to experience chronic absenteeism and are subjected to punitive disciplinary measures, such as suspensions and expulsions. Collectively, these studies emphasise the systemic nature of absenteeism among learners with disabilities and the urgent need for inclusive and equitable policy responses.

### Disengagement

Disengagement, which prevents learners from staying focused on academic tasks, is considered a persistent challenge to effective school learning (Leepo 2015, cited in Ahmed 2025). This issue is particularly pronounced among learners with learning disabilities, who often face difficulties sustaining academic motivation and confidence. Kausik and Hussain (2023) investigated the impact of inclusive education on the academic motivation, self-efficacy, and well-being of learners with learning disorders by comparing three groups: those enrolled in special schools, inclusive schools, and those not attending school. Their findings revealed that learners without learning disabilities consistently demonstrated higher levels of self-efficacy, academic motivation, and overall well-being compared to their peers in inclusive settings. Learners with learning disabilities were found to struggle with academic persistence, often because of repeated failures and limited success, which in turn undermines their belief in their academic abilities. Ahmed (2025) further explains that diminished self-efficacy and motivation can significantly impair a learner’s capacity to engage meaningfully in classroom activities, frequently resulting in behavioural challenges and underachievement. Similarly, Oliveira and Lathrop (2022) identify related difficulties, such as incomplete schoolwork, reduced classroom participation, and cognitive processing struggles. Within the context of special schools, these findings underscore the considerable challenge teachers face in fostering motivation and active participation among learners with learning disabilities.

### Other causes of indiscipline

Social media, family dynamics, peer influence, and socioeconomic conditions collectively shape learner behaviour and contribute significantly to school indiscipline. Potokri and Lumadi (2025) contend that learners are conditioned to violence by continual exposure to hostile content on television and social media. They found that many adolescents are raised in contexts where violence is normalised, not only within their communities but also through the media they engage with, resulting in the perception of aggressiveness as a valid and successful method for attaining their objectives. Participants attributed media exposure, particularly to violent, sexual, or unethical content, as a significant catalyst for learners’ indiscipline. This viewpoint is corroborated by Matimba (2023), who indicates that more than 89% of such activities can be ascribed to media impact. Matimba asserts that this style of content undermines respect for authority and frequently romanticises rebellion, which participants believe fosters bad attitudes and behavioural issues among learners. These findings underscore the influence that media may have on student behaviour and indicate that ongoing exposure to detrimental content may exacerbate disciplinary issues in schools.

According to Wolhuter and Van der Walt (2020), inconsistent parenting, characterised by inadequate supervision and poor role modelling, often leads to emotional instability and resistance to authority. This view is supported by Rachel, Roman and Donga (2022), who argue that ineffective parental involvement fosters behavioural problems, particularly when parents fail to reinforce expectations at home. Furthermore, Obadire and Sinthumule (2021) contend that children exposed to violence or substance abuse in their household environments often replicate such behaviours at school, as aggression becomes normalised. Similarly, Posey-Maddox and Haley-Lock (2020) emphasise that a lack of parental engagement in education undermines teachers’ efforts to maintain discipline, as learners receive minimal reinforcement of school values outside the classroom. Moving beyond the family unit, Lazaratou et al. (2017) report that poverty is associated with increased aggression and emotional distress, while Paulson et al. (2022) explain that chronic stress caused by socioeconomic hardship weakens learner–teacher relationships, further increasing the likelihood of indiscipline. Moreover, peer pressure serves as a powerful force during adolescence. As Laursen and Veenstra (2021) explain, learners often conform to group norms to gain acceptance, even when those norms promote misconduct. Veenstra, Dijkstra and Kreager (2018) and Adeniyi and Jinadu (2021) add that popular peers frequently set behavioural standards, sometimes encouraging defiance or bullying to assert dominance. According to Lunga (2020), this tendency to emulate influential group members means that peer dynamics can either promote or undermine discipline, depending on the values held within the group. Therefore, school indiscipline is rarely the result of a single factor, but rather the outcome of intersecting familial, economic, and social influences.

## Types of disabilities and indiscipline

### Learning disorder

Therese et al. (2023) assert that learners with learning disorders often display social and intellectual deficiencies, which increase their vulnerability to academic failure or dropout. These challenges may lead to the perception of such learners as disruptors, which obstructs the recognition of their underlying difficulties. Heudes (2022) suggests that children with externalising or internalising disorders are particularly prone to inappropriate peer victimisation because of their behaviour. Individuals with these conditions may exhibit aggressive or distressing actions, potentially leading to negative peer interactions. This heightened vulnerability, coupled with disruptive behaviours, further exacerbates the likelihood of these children disturbing the tranquillity of the classroom, contributing to disciplinary issues. Such actions undermine their academic progress and disrupt the broader learning environment, as teachers and peers may struggle to manage these behavioural challenges, thereby increasing the risk of disengagement and further adverse outcomes.

### Visually impaired and blind

Mohamed, Abdelrahem and Ahmed (2019) describe visual impairment as a condition marked by reduced visual capacity that cannot be corrected through conventional surgery or contact lenses. This condition encompasses a range of impairments, from partial vision loss to complete blindness. Learners with visual impairments often exhibit behavioural issues, such as peer rejection, maladjustment, aggressiveness, physical altercations, and antagonism, all of which are indicative of indiscipline in educational settings (Fathermeh 2012, cited in Mohamed et al. 2019). Ghorbaninejad et al. (2020) highlight several factors that contribute to the emergence of aggressive behaviours in individuals with visual impairments. These include negative societal perceptions, heightened dependence on parental figures, and insufficient supervision or support. These challenges, when combined, can undermine the development of these learners, potentially disrupting the classroom environment. As such, the behaviours stemming from their condition may pose a significant threat to creating a conducive learning environment, fostering indiscipline and further hindering their educational experiences and integration.

### Hearing impairment and deaf

Dash (2000, cited in Hameed, Irshad and Mushtaq (2023) observed that hearing impairment is a form of damage or defect to the auditory process, potentially leading to a range of hearing loss, from mild to profound. Shoko (2024) highlights that poor communication skills can contribute to behaviours that may be perceived as misconduct. This suggests that schools accommodating learners with hearing impairments often encounter challenges because of the insufficient communication skills of staff and administrators, which hinder effective interaction with deaf learners. Adeniyi, Olufemi-Adeniyi and Raheem (2021) further observe that adolescents with hearing impairments are prone to a range of behavioural issues, including restlessness, distractibility, irritability, hyperactivity, aggression, a lack of persistence, excessive sobbing over minor irritations, and tendencies to be shy or distrustful of others. These difficulties, arising from auditory deprivation, may exacerbate the likelihood of disruptive behaviours. Awan et al. (2024) also found that children and adolescents with hearing impairments are more vulnerable to behavioural problems than their typically hearing peers. Consequently, these conditions may significantly threaten the establishment of a conducive learning environment. The challenges in communication and behavioural regulation can contribute to increasing indiscipline, as teachers and peers may struggle to effectively address and accommodate these learners’ unique needs.

### Attention deficit hyperactivity disorder

Attention deficit hyperactivity disorder is a condition defined as difficulties in attention, impulsivity, and hyperactivity (Celis et al. 2023). Alhwaiti (2022) states that ADHD is a mental illness characterised by impaired focus and attention, which may be provoked by causes such as the inability to remain seated, hyperactivity, and forgetfulness. These traits may affect learners’ behaviour in schools. For example, one may participate in unsuitable discussions with peers or express opinions without seeking consent. An excessively active individual may struggle to remain sitting because they often fidget with things, rock their seats, and incessantly tap their feet or hands (DuPaul & Stoner 2003, cited in Bolinger et al. 2020). Kusmawati, Fahrurrozi and Supena (2023) assert that the attention span of learners diagnosed with ADHD presents a significant barrier in the teaching and learning process, necessitating specialised instructional strategies. Such a short attention span may also contribute to learners opting for other activities that may constitute indiscipline.

### Autism spectrum disorder

The American Psychological Association (2013, cited in Pienaar & Dreyer 2024) defines ASD as a neurodevelopmental disorder impacting children in early childhood. This may arise from deficiencies in social interaction skills, including challenges in recognising people and establishing eye contact. They may also display indications of confined and repetitive activities. Erasmus, Kritzinger and Van Der Linde (2019) conducted a study investigating the profiles of public and private autism-specific schools in Gauteng province. The study found that learners with ASD frequently exhibit behavioural difficulties that are significantly worse at school than at home. These issues may include cognitive inflexibility, repetitive play behaviours, and a lack of awareness about the consequences of their activities. These behaviours are some of the incidents of indiscipline impacting the teaching and learning process.

### Intellectual disability

The World Health Organization (2022, cited in McKenzie et al. 2024) defines intellectual disability as a neurodevelopmental disease that impairs people’s cognitive and adaptive functioning. The American Association of Intellectual and Developmental Disabilities (2021, cited in Westrop et al. 2024) and Saini et al. (2024) argue that persons with intellectual impairments generally struggle with adaptive and cognitive capacities throughout developmental stages. Nicholls, Hastings and Grindle (2020) found, in their study on a modified version of the Behaviour Problems Inventory, that children with intellectual impairments are prone to displaying problematic behaviours, including destructive and aggressive actions. The results indicated that these learners are likelier to display traits teachers may perceive as challenging and threatening a conducive learning environment.

## Problem statement

Instances of indiscipline have been observed in special schools (Anderson 2020; Idris & Badzis 2017; Shoko 2024; Van Der Linde 2019), and learners in such schools experience severe prejudice and worse penalties compared to their peers without disabilities (Gage et al. 2021). Research shows that compared to learners in mainstream schools, these learners are more likely to be suspended temporarily from the schools (Anderson 2020). The results led Browning (2019) to assert the need to identify strategies and procedures to assist teachers and administrators in special schools in managing disciplinary concerns. Van Der Linde (2019) has advocated for more studies addressing indiscipline in special schools, citing a paucity of studies within the South African setting and other nations (Florez 2021). Le Grange (2021) asserts that teachers in special schools have many problems with learners, which affect their effectiveness in classroom management and maintaining a good attitude. However, as Van Der Linde (2019) noticed, the limited research focusing on discipline in South African special schools required attention. To contribute to the body of knowledge in the field, this study explored the disciplinary issues the teachers experience in special schools and the contributing factors. The following question guided the article:

*What disciplinary issues do teachers experience in special schools, and what are they attributing those to?*

## Theoretical framework

### Bronfenbrenner’s ecological systems theory

This study was anchored in Bronfenbrenner’s Ecological Systems Theory to facilitate a comprehensive understanding of the indiscipline issues teachers experience in special schools. The theory posits that a child develops within a nested ecosystem of social, institutional, and cultural systems, where interactions across multiple levels collectively shape behaviour and development (Maniram 2015). This suggests that learners’ behaviour is influenced not only by immediate environments such as the home and school but also by broader societal and community structures.

By applying this framework, the study moved beyond attributing indiscipline to individual learner traits or disabilities. Instead, it illuminated the complex interplay of relational, environmental, and systemic factors that contribute to learner behaviour (Lansey, Burnette & Ryndak 2023; Maniram 2015). These included family involvement, classroom dynamics, socioeconomic conditions, and prevailing societal attitudes towards disability and discipline.

Bronfenbrenner’s assertion that disruptions or stressors in one ecological layer can reverberate across the system was instrumental in interpreting teachers’ experiences and understanding the experiences regarding indiscipline within special school contexts. The study utilised three fundamental system levels to examine the dynamics of learner indiscipline:

### Microsystem: Classroom interactions and direct relationships

At the microsystem level, the theory proved pertinent in enhancing comprehension of how the immediate environment, including classrooms, peer interactions, and familial relationships, influences learner behaviour.

### Mesosystem and exosystemic: Familial support and institutional obstacles

At the mesosystemic level, Bronfenbrenner’s theory offered a valuable framework for analysing the interaction between home and school environments, particularly in understanding how varying degrees of family involvement and caregivers’ competence in addressing disability-specific needs, such as communication support, contribute to learner behaviour. In addition, the theory provided a structured lens for interpreting the indirect influence of institutional constraints on learner conduct by highlighting how these limitations shape the broader teaching and learning conditions within special schools.

### Macrosystem: Cultural perspectives, disparities, and societal influences

At the macrosystem level, the theory enhances comprehension of cultural norms, socio-political institutions, and social attitudes, and their influence on learner behaviour.

Grounding this study on Bronfenbrenner’s Ecological Systems Theory proved essential in unpacking the multifaceted nature of indiscipline in special schools. It also enabled the study to draw nuanced conclusions, considering the multiple interrelated layers that influence learner behaviour.

## Research methods and design

This study employed an interpretive qualitative approach and a multiple case study design in two purposively selected special secondary schools. Data were collected through semi-structured interviews with four teachers and two heads of departments.

Participants for the study were recruited with the approval of the school principal, who authorised the research by signing a request letter. This letter provided key details about the study, and informational leaflets were distributed to all staff members. Furthermore, a verbal presentation was conducted to clarify the nature of participation and highlight the study’s importance, nature of involvement and the required number of participants. The principal coordinated the scheduling of this session. Participation was open to both teachers and School Management Team (SMT) members, as all roles, as defined by the *Personnel Administrative Measures* (Republic of South Africa [RSA] 2022), involve teaching responsibilities. The first three staff members who expressed interest were selected for interviews, which were arranged by the researchers on the designated day. Informed consent was obtained from all participants, allowing the researchers to record the interviews. Semi-structured interviews were used to generate data. A combination of open-ended and closed-ended questions during interviews allowed teachers to share their in-depth experiences (Kallio et al. 2016).

An inductive content analysis approach was used to analyse the transcribed data. This method allowed the data to guide the development of themes derived directly from the data rather than predetermined by the researcher (Vears & Gillam 2022). Initially, the researchers reviewed the data to gain a preliminary understanding, followed by open coding to break down the data into smaller, significant segments. These codes were then organised into broader themes or groups based on their similarity and importance without relying on predefined categories. The researchers continuously refined these themes through ongoing comparison with the data to ensure they accurately represented the participants’ experiences.

The final stage of the analysis involved presenting the findings, highlighting the main themes and interpreting them concerning ecological theory, supported by direct participant quotes. Direct quotations were included to enhance the study’s credibility to provide concrete examples and substantiate the researchers’ interpretations. This ensured that the results were grounded in the participants’ experiences rather than the researchers’ biases or assumptions.

Table 1 and Table 2 represent the demographic details of the research sites and the participants for schools A and B.

**TABLE 1:** Details of the sampled secondary schools.

Name of school	Number of principals	Number of deputy principal	Number of departmental heads	Number of teachers	Number of learners
School A	1	1	3	23	79
School B	1	2	8	36	336

**TABLE 2:** Participants’ biographic details.

Pseudonyms	School	Position	Gender	Teaching experience	Highest qualification
P1	A	Teacher	F	35	B.Ed.
P2	A	Teacher	F	31	B.Ed. Honours
P3	A	Teacher	F	13	B.Ed.
P4	B	DH	M	22	B.Ed. Honours
P5	B	DH	M	13	B.Ed. Honours
P6	B	Teacher	F	19	ACE

B.Ed., Bachelor of Education; ACE, Advanced Certificate in Education; F, female; M, male; DH, departmental head.

### Ethical considerations

The study received ethical approval from the Free State Department of Education and the University of Free State Ethics Committee. The approval was authorised on 21 August 2024 with the ethical clearance number UFS-HSD2024/1446.

## Results

### The disciplinary issues the teachers experience in special schools

The participants indicated that they experience indiscipline through learners’ disengagement towards schoolwork, bullying, and absenteeism (Table 3).

**TABLE 3:** Summary of inductive content analysis procedure.

Main Theme	Sub-theme	Codes	Illustrative quotes
Disciplinary issues that teachers experience in special schools	Disengagement	Learner refusal to complete tasks Passive resistance to schoolwork Negative learner disposition	‘The type of indiscipline that I encounter is learners not doing their work. Laziness’. ‘… they do not want to do their work; they do not want to be here’. (P1, A, teacher, F, 35)
	Bullying	Physical altercations among learners Gossip-driven conflict Hierarchical learner interactions	‘Physical fights that break out from time to time’. ‘… gossiping contributes … girls bad mouth each other …’ ‘Everybody has a way of exerting himself … a desire to dominate others’.
	Absenteeism	Learner absenteeism because of substance use Sporadic school attendance Instructional disruptions	‘Mostly *ba abscond* [they] … because of substance abuse’. ‘Some go for a long time, and then they come back again …’ (P2, A, teacher, F, 31)
Factors that contribute to indiscipline in special schools	School contextual factors:		
	Curriculum pressure	Academic workload overwhelming learners Learner frustration because of curriculum mismatch Avoidant misbehaviour	‘… homework, and strenuous activities … frustration on the learner …’ ‘They cannot cope … try to hide this behaviour …’ (P5, B, DH, M, 13)
	Teachers’ leniency	Over-sympathetic disciplinary approach Reluctance to enforce rules Lack of strong consequences	‘… we are soft on them because of their background …’ ‘We can’t suspend them …’ (P4, B, DH, M, 22)
	Out-of-school context:		
	Family	Emotional neglect at home Disrupted family structures Search for emotional validation	‘Maybe they grow up in a household where the parents are absent …’ ‘… broken home … they want to belong …’ (P3, A, teacher, F, 13)
	Communication barriers	Parent-child language disconnect Lack of home-based discipline foundation Deaf learners’ communication limitations	‘… because of communication problems, which is sign language …’ ‘… child would not have had communication or discipline from the time while she was still young …’ (P4, B, DH, M, 22)
	Social grant support influence	Learner financial autonomy undermining discipline Parental disempowerment Exploitation of grants	‘SASSA Grants … learners are now independent …’ ‘… learner have taken their SASSA card …’ (P5, B, DH, M, 13)
	Racial dominance	Racial-based social grouping Racial bullying and exclusion Persistence of racial stereotypes	‘Sotho girls against the Afrikaans girls …’ ‘Afrikaans white people get bullied …’ (P1, A, teacher, F, 35)
	Learner factors:		
	Peer influence	Peer pressure to attain status Materialistic competition Romantic exploitation among learners	‘Peer pressure … social grant cards … buy others for romantic benefits …’ (P5, B, DH, M, 13)
	Disability-based behaviour	Learning challenges because of disability Communication gaps linked to deafness Frustration-induced misbehaviour	‘Sign language might be the major …’ ‘They do not understand what they are reading … contributes to indiscipline …’ (P5, B, DH, M, 13)

#### Disengagement

Data indicated that learners in special schools often display attitudes characteristic of learners with disabilities, although not being so, by showing reluctance to engage in their academic responsibilities and exhibiting disengagement. A participant from school B had the following to share:

‘The type of indiscipline that I encounter is learners not doing their work. Laziness.’ (P4, B, DH, M, 22)

A participant from school A added:

‘Yeah. It is more with homework and learning. Remember, although the kids are in a special school and they do not have physical disadvantages, you can see that the attitude is more of a disadvantage than having a physical disadvantage. They do not want to do their work; they do not want to be here.’ (P1, A, teacher, F, 35)

#### Bullying

The participants also indicated that they experience indiscipline in the form of bullying. According to the experiences of the participants, bullying manifests in two different forms in these types of schools. It is through physical fights influenced by gossip and other learners’ desire for dominance. In terms of bullying through physical fights, A participant indicated:

‘Then, there are also, of course, physical fights that break out from time to time.’ (P3, A, teacher, F, 13)

Another participant shared:

‘Sometimes, gossiping contributes. That is many discipline problems of the girls. They tend to bad mouth each other, which turns into a fight.’ (P1, A, teacher, F, 35)

Regarding bullying that comes because of the quest for dominance, one participant indicated:

‘I mean, it would not have been normal if there had been no bullying. Everybody has a way of exerting himself and a desire to dominate others. I mean it’s a normal thing. That is why there’s got to be educators and parents to discipline and to guide all the time. There is, but it has got to be managed and disciplined.’ (P4, B, DH, M, 22)

#### Absenteeism

The participants additionally stated that they encounter indiscipline in the form of learners who are briefly absent from school for various reasons and then return to resume their studies. As a result, they indicated that the teaching process becomes disorganised and lacks consistency. One participant stressed:

‘Mostly ba absconder (they abscond) because of substance abuse. So, there is absenteeism. Some go for a long time, and then they come back again. You must start from scratch in terms of teaching when they come back. We are re-admitting learners throughout the year.’ (P2, A, teacher, F, 31)

The quotation suggests that educators in special schools face several forms of indiscipline stemming from diverse social interactions and the societal norms that shape everyone’s development.

### Factors that contribute to indiscipline in special schools

The participants attributed indiscipline in special schools to schools, out-of-school, and learner factors (Table 3).

#### School contextual factors

The data revealed that curriculum pressure and teachers’ leniency are school contextual factors contributing to indiscipline in these types of schools.

**Curriculum pressure:** The participants revealed that learners misbehave because of the pressure that they feel because they are doing the mainstream curriculum, which does not allow learners with special needs time to cope or adapt; some of them are misbehaving because of their frustration. A participant highlighted:

‘Perhaps a lot of work, homework, and strenuous activities that every learner should do because we do a main curriculum. That may put frustration on the learner given their limitations as well in terms of the disability. So, the school system in itself puts tremendous pressure on learners, especially in higher grades. And that alone can cause an outburst in the learner.’ (P5, B, DH, M, 13)

Another participant also stated:

‘I think many kids cannot cope. They have a backlog in education, and they cannot cope. Because they cannot cope, they try to hide this behaviour by being busy with other things that make them look cool or tough. That is why they misbehave.’ (P3, A, teacher, F, 13)

**Teachers’ leniency:** The participants also revealed that the school influences the school contextual factors in various ways, such as being lenient on consequences for learners. They alleged that learners with special needs misbehave knowing that there are no consequences for them because of their disability. One participant elaborated:

‘Yeah, like I slightly indicated, It’s so difficult to apply consequences. You know, they can see that we are considerate. We are soft on them because of their background, because they have a disability. Therefore, we think more along those lines. We are unable to be very strict. We can’t suspend them; we can’t dismiss them. All those types of things. We suspend at extreme measures level.’ (P4, B, DH, M, 22)

#### Out-of-school context

According to the data, indiscipline in schools is also caused by various external factors outside of the school setting. These include family, communication barriers, social grant support system, social media and race.

**Family:** The participants indicated familial influences learners to be indiscipline as some seek attention and belonging at school when they do not receive it from home. A participant stated:

‘Maybe they grow up in a household where the parents are absent. I think that has got a huge influence. I think sometimes kids feel unloved, which causes them to act out and to do things. So, they are looking for love in all the wrong places. They are looking for acceptance. The people who sometimes accept you are in the same situation.’ (P3, A, teacher, F, 13)

Another participant from school A added:

‘The challenges outside the school are normally family. This broken family absenteeism of fathers. The grandma is raising the kids. They do not have a sense of belonging because they come out of a broken home; they want to belong, and they want attention.’ (P1, A, teacher, F, 35)

**Communication barriers:** Participants indicated that some parents do not use sign language, hindering their ability to convey appropriate values from birth and complicating teachers’ work regarding behaviour when these learners begin their education.

One participant elaborated:

‘When the kids come from their homes, you know, they don’t have good relationships with their parents because of communication problems, which is sign language. So that has got a serious problem because the parents themselves are also lenient to these kids because they are unable to communicate with them, so that on its own, the child would not have had communication or discipline from the time while she was still young. So, when he or she starts school year, we start there. And sometimes to be disciplined by somebody that is not your parent is not easy.’ (P4, B, DH, M, 22)

**Social grant support influence:** Participants noticed that learners with special needs receive social grants, which is aimed to help them with their basic needs. They alleged that parents no longer take part in their learner’s education and behaviour because these learners take their cards into which the money is deposited to school. They also indicated that the social grant support sometimes is used by other family members for their own benefits instead of intended recipients, leading to the development of unacceptable behaviour. A participant shared:

‘I think the main is the South African Social Security Agency (SASSA) grants which directly affects education. Because the learners are now independent, they are mothers and fathers of their own. They must take accountability for their school fees. Now, even when you call parents for ill-discipline of the learner, the parent will not necessarily come, so it’s quite disturbing in the special schools given how the parents relate with the learners with special needs and social grants in between.’ (P5, B, DH, M, 13)

Another participant further elaborated:

‘Because most of our learners are orphans, so, we find that they are staying with their uncles. And then you know that they are receiving the grants. Then, the people staying with the kid will use a grant for themselves. The child will start changing in terms of behaviour. Immediately, you do the research, you find the root-cause.’ (P6, B, teacher, F, 19)

One participant also added:

‘And parental involvement has been contaminated by SASSA grants. Because when you speak to the parent about the responsibilities in their learner involvement, they will say the learner have taken their SASSA card. So, when they have taken their SASSA card, then it suggests that the learner is independent, and has got the money.’ (P5, B, DH, M, 13)

**Racial dominance:** The data indicated that special schools accommodate learners from varied racial backgrounds because of their limited numbers. As a result of certain races’ attempts to control others, they alleged that racial abuse between learners leads to disobedience.

One participant highlighted that:

‘I think, basically, the girls come from various groups. Afrikaans English. Sometimes, they form groups in society or in school. So, the Sotho girls against the Afrikaans girls or against the Tswana girls. It is still there; we experience it. Yes! That inherited racism is still there. I can say the Afrikaans, not the Afrikaans coloured girls, but the Afrikaans white people get bullied. They get bullied a lot. Yeah, because there are only a few in this school.’ (P1, A, teacher, F, 35)

Indiscipline arises because of different external factors that impact learning environments. It is evident from the findings that learners living in environments that are characterised by high levels of parental influence, socioeconomic status, substance abuse, and a poor home environment tend to exhibit an increased tendency to exhibit indiscipline in special schools. Moreover, the data show that special schools are characterised by huge diverse races because of accommodating learners with unique challenges; for that, there is a race diversity that leads to derogating comments, causing indiscipline in schools.

#### Learner factor

The participants alleged that some of the issues encountered by learners in special schools could be attributed to the influence of peers and their disability, which could include non-compliance with school regulations.

**Peer influence:** Peer influence at special schools is attributed to access to social grant money, which enables individuals to influence others in many ways to serve them in a desire for belonging or to retain friendships. One participant stated:

‘Peer pressure amongst themselves is common because others have got what I mentioned earlier as social grant cards on their own. So, if they have R1000, whatever amount of money to themselves, they can buy clothes, they can buy cell phones. They can do so many things. So, peer pressure is quite rough, and others will end up serving others because of money. So, you find that then others will now lead. Others will also buy others for romantic benefits. So, you find those activities happening because of social or peer pressure amongst themselves.’ (P5, B, DH, M, 13)

**Disability-based behaviour:** The participants mentioned that learners misbehave because of their disability, such as communication difficulties and a lack of understanding from their teachers and peers, which leads them to start misbehaving because of their frustration. A participant from school B explained:

‘It frustrates when one does not understand the other. Communication barriers are one of the factors that cause divorce, even in marriages. That factor leads to learners’ misbehaviour or wrong conception of what they do because of the language barrier. Sign language might be the major. It even gets worse if the teacher does not have the skill of sign language and the learners do not understand, or if the learner comes from the mainstream school into the school of the deaf and they cannot speak, cannot even hear the verbal language. So, they are in either of the two worlds: the deaf are not in the hearing world. So that is what causes that outburst. It happens.’ (P5, B, DH, M, 13)

Another participant elaborated that:

‘Yeah, education for deaf learners is not standardised in a way that will be receptive to them. For example, sign language is a new language to them. It has the rules they have to follow. That on its own is new even to educators. When you look at the background of deaf people, their language is not written or spoken. So, it becomes a problem when you ask them to write or read. That on its own is a bearing on them. They would feel like they do not need to do the work. They do not understand what they are reading. They just come to school and go back because. That is what we are doing. We do the work for them. This may contribute to indiscipline because they somehow feel they do not benefit from it.’ (P4, B, DH, M, 22)

The excerpt indicates that microsystems, including family and school interactions, impact indiscipline in special schools. The implication is that environment, particularly familial background, type of social system available to learners (mesosystem), teachers’ beliefs towards discipline, which influenced their leniency (macrosystem), significantly impact indiscipline in these schools.

## Discussion

This study’s findings underscore a complex interplay of disciplinary challenges encountered by teachers in special schools, illustrating a nuanced array of causes that lead to student indiscipline. Disengagement, bullying, and absenteeism are intricately connected to several environmental, family, and societal factors, all of which correlate with Bronfenbrenner’s Ecological Systems Theory. This theory highlights the various levels of impact on a child’s development, with elements from the microsystem (individual interactions) to the macrosystem (broader social circumstances) affecting behaviour.

As noticed in this study, disengagement is evident in learners’ unwillingness to finish or do assignments, which undermines the educational atmosphere. This issue may be somewhat associated with the learners’ disability, but it is also affected by an absence of adequate structure and participation within the learning environment. The study revealed that teachers at special schools frequently used a lenient approach, perhaps in response to the distinct conditions of the learners. This tolerance, albeit well-intentioned, may unintentionally foster a lack of accountability, permitting complacency to endure. Moreover, the curriculum offered, which is similar to that of mainstream schools, may not adequately cater to the specific needs of learners with disabilities, so it may intensify their disengagement and lack of motivation. Maphalala and Adigun (2024) observed that the adaptation of the curriculum for learners with disabilities is often beyond the teachers suggesting a need for relevant stakeholder intervention in this regard.

Bullying is a common problem in special schools, arising from a confluence of variables (Mareš 2018; Robinson et al. 2023). This study found that peer pressure leads learners with impairments to seek control over their peers, frequently instigated by rumours and conflicts. This conduct signifies underlying difficulties of social power and control. The presence or absence of social support networks is crucial in this context. Individuals without confidence or abilities may turn to bullying to attain social dominance (Laursen & Veenstra 2021). The findings of this study revealed that the racial dynamics inside the school exacerbate this issue, as learners from various ethnic origins strive to assert dominance or marginalise their peers. This racial tension signifies more extensive social problems that permeate micro-level interactions among learners, enduring despite the nation’s democratic structure. This is apparent in the historical backdrop and present circumstances, when certain political parties declined to establish a National Government of Unity owing to racial disparities. Societal divisions are reflected in schools, since they frequently mirror the community. Franco, Durkee and McElroy-Heltzel (2021) emphasised the detrimental effects of racial discrimination on conduct and mental health, observing that victims of racism often endure rage and dissatisfaction. Feelings of resentment may exacerbate behavioural problems in special schools, especially among learners who perceive marginalisation by other racial groups.

Absenteeism disrupts the continuity of the educational process. The study found that learners frequently skip many school days and then face overwhelming assignments, resulting in frustration and disengagement. The teachers are placed in a challenging predicament of accommodating these learners by re-instructing material, further interrupting the educational progression for the remainder of the class. Rafa (2017) observed that learners with impairments are 1.4 times more likely to be chronically absent compared to their counterparts without disabilities. This absence can be attributed to external issues, such as inadequate familial support noticed by Rachel et al. (2022), which in this study is attributed to certain families’ disregard for their children’s needs, and the misuse of social assistance money for their own benefits rather than those of the child. This neglect might result in the learners feeling disappointed and demotivated, impacting their behaviour both in school and outside of it. Wolhuter and Van der Walt (2020) assert that parents play a significant role in shaping their children’s attitudes towards behaviour in school. This absence reflects broader structural challenges that may be beyond the school’s control.

From an ecological systems perspective, these disciplinary issues are not isolated; rather, they are the result of an intricate web of interactions between various environmental layers. At the microsystem level, the teacher–student relationship and classroom dynamics are crucial in shaping student behaviour. The leniency of teachers, because of their empathy towards the unique challenges faced by learners with disabilities, can inadvertently create an environment where indiscipline is tolerated. However, this leniency must be understood within the context of the mesosystem, the broader connections between the school and the family. The study reveals that some families fail to provide the necessary support for their children, resulting in unmet emotional and developmental needs that manifest as behavioural issues in school. The inability of families to impart values to their children, especially in the absence of sign language proficiency, contributes to misunderstandings and, at times, misbehaviour. This lack of clear communication between the school and the family creates an environment where disciplinary problems can thrive. Matimba (2023) found that children’s education about morals is being compromised by the failure of parents to teach them proper values. Shoko (2024) also found that being unable to communicate effectively may result in issues perceived as misbehaviour.

Furthermore, the exosystem level, which refers to the indirect influences on a child’s immediate environment, is also a significant factor. Social support systems, including financial aid or peer pressure stemming from money, were found to play a role in encouraging misbehaviour. Learners who receive social support funds may use this money to influence others, fostering peer pressure that leads to indiscipline. This issue is compounded by racial tensions within the school, which can create an atmosphere of competition among learners, exacerbating bullying and dominance struggles.

Finally, at the macrosystem level, broader societal influences such as racial inequality and societal views on disability shape how individuals within the school interact with one another. The fact that special schools in the country cater to a mix of different races can lead to power struggles, with some learners trying to assert dominance over others based on racial differences. This social dynamic contributes significantly to the bullying observed in the study.

## Conclusion

The disciplinary issues observed in special schools, such as disengagement, bullying, and absenteeism, cannot be fully understood in isolation. They result from a complex interplay of factors within the school environment, the family context, the social support systems, and broader societal influences. By applying Bronfenbrenner’s Ecological Systems Theory, we may argue that these issues are not merely individual challenges but are shaped by multiple layers of influence. Teachers’ leniency, the curriculum’s lack of adaptation to learners’ needs, family neglect, and social pressures all contribute to the indiscipline observed in these schools. Addressing these issues requires a holistic approach that considers all these layers and their interactions, ensuring that learners with disabilities receive the support they need in their educational and personal development.

### Recommendations

- Establish peer mentoring programmes to promote positive behaviour and reduce bullying through social support. The study revealed that bullying, often driven by gossip, dominance, and racial tensions, is a common disciplinary issue. To address this, schools should implement peer-led conflict resolution initiatives and conduct cultural diversity workshops that foster understanding and inclusion. A clear anti-bullying policy must be enforced, while learners should be encouraged to engage in activities that build empathy and social cohesion. Additionally, partnering with local NGOs and community leaders may support racial reconciliation dialogues and restorative practices within the school community.
- Enhance teacher training to equip educators with effective classroom management strategies and skills to address behaviours such as disengagement, absenteeism, bullying, and challenges related to disabilities. The study revealed that disengagement often stems from frustration with an inflexible curriculum and attitudinal issues. Regular training should focus on differentiated instruction, trauma-informed teaching, and motivational techniques. Given the communication barriers identified, all educators should receive sign language training. Schools should invest in communication aids and recruit or train assistants fluent in sign language. Visual aids and alternative communication methods should also be incorporated to support understanding.
- Strengthen home-school partnerships through consistent communication and parenting workshops to address behavioural issues and support family dynamics. The study revealed that many learners come from homes with absent or uninvolved parents, often due to broken families or dependence on social grants. To improve involvement, schools may host workshops on positive parenting and the importance of family support in special needs education. A home-school liaison team can facilitate better communication and conduct home visits where necessary. Additionally, schools should collaborate with the Department of Social Development to ensure that grants benefit learners. Working with social workers to promote financial responsibility is also vital. Finally, economic literacy and peer mentoring programmes may help reduce the impact of monetary peer pressure.
- Adapt the curriculum to be more inclusive by ensuring it meets diverse learning needs and reduces frustration. The study found that curriculum demands often overwhelm learners in special schools, leading to disengagement and disruptive behaviour. The education authorities should revise the curriculum to allow flexible pacing and alternative assessments. Schools should also implement and regularly monitor Individualised Education Plans (IEPs) tailored to each learner’s needs.

### Limitations and further research

The study was limited by scope, as it only investigated one province, one district, and a limited number of special schools. Also, because it only gathered a small sample of teachers, future research may investigate how social grants affect learners’ behaviour and their parents’ involvement in educational institutions. Other studies may focus on each disability in depth regarding its influence on indiscipline, as this study generalised all the types of disabilities in special schools. In addition, future research may analyse the best practices of professional development programmes to enhance special education teachers’ capacity to deal with disciplinary issues.
